# Root–Canopy Coordination Drives High Yield and Nitrogen Use Efficiency in Dryland Winter Wheat

**DOI:** 10.3390/plants15010153

**Published:** 2026-01-04

**Authors:** Meng Li, Limin Zhang, Yuanxin Li, Yunxuan Cao, Yueran Zhang, Zhiqiang Gao, Dongsheng Zhang, Wen Lin

**Affiliations:** Key Laboratory of Sustainable Dryland Agriculture of Shanxi Province, College of Agriculture, Shanxi Agricultural University, Taiyuan 030031, China; 20232168@stu.sxau.edu.cn (M.L.); z20223097@stu.sxau.edu.cn (L.Z.); 20220101012@stu.sxau.edu.cn (Y.L.); 20233732@stu.sxau.edu.cn (Y.C.); 20220101023@stu.sxau.edu.cn (Y.Z.); gaozhiqiang1964@sxau.edu.cn (Z.G.)

**Keywords:** grain filling, dry matter accumulation, water use, nitrogen uptake, cultivar differences

## Abstract

Improving yield and nitrogen-use efficiency (NUE) is essential for dryland winter wheat. We hypothesized that cultivars classified as high-yield and high-efficiency (HH) achieve superior performance through coordinated root–canopy traits that enhance water and nitrogen acquisition, sustain post-anthesis photosynthesis, and maintain assimilate and nitrogen remobilization. A two-year field experiment was conducted using ten regionally representative cultivars, which were grouped into HH, high-yield and low-efficiency (HL), low-yield and high-efficiency (LH), and low-yield and low-efficiency (LL) types based strictly on grain yield and NUE. Measurements included yield components, grain-filling, dry matter accumulation and partitioning, soil water use, nitrogen uptake and remobilization, and root–canopy structural traits. HH increased yield by 41.5% and water-use efficiency by 24.1% relative to LH, supported by denser shallow roots, moderate deeper-root development, higher leaf area index, and more compact canopies. HH also exhibited stronger post-anthesis dry matter and nitrogen translocation, resulting in a larger grain number per unit area and improved sink capacity. Correlation analyses further demonstrated positive associations among root–canopy traits, water and nitrogen dynamics, and yield formation. These results support the hypothesis that a coordinated root–canopy structure underlies the superior yield and NUE performance of HH cultivars in dryland systems, providing a physiological basis for cultivar improvement.

## 1. Introduction

Winter wheat is a crucial staple crop in the rainfed agricultural systems of northern China. However, its growth and productivity in this region are constrained by multiple challenges, such as the uneven spatiotemporal distribution of precipitation, high atmospheric evaporative demand, and severe soil erosion. Consequently, wheat cultivation here faces the dual limitations of water deficit and nitrogen deficiency [1], leading to suboptimal yields with considerable year-to-year fluctuations. This has emerged as one of the key factors limiting regional grain production capacity [2]. Therefore, enhancing the water use efficiency (WUE) and nitrogen use efficiency (NUE) of winter wheat to achieve high and stable yields under these resource-limited conditions represents a central scientific challenge for the sustainable development of dryland agriculture [3].

The formation of crop yield is fundamentally an integrated process encompassing dry matter production, accumulation, and remobilization. Water availability significantly influences photosynthetic rates and assimilate accumulation [4], while nitrogen (N) regulates leaf photosynthetic apparatus and source–sink dynamics, thereby affecting carbon partitioning patterns [5,6]. Previous studies have demonstrated that variations in winter wheat yield under rainfed conditions are governed not only by inherent photosynthetic potential but also by key physiological traits, including root water absorption depth, post-anthesis assimilate remobilization capacity, and N remobilization efficiency [7,8]. However, most studies have focused predominantly on single factors (e.g., photosynthesis or grain-filling), lacking systematic comparisons among different yield-performing groups within the context of water–nitrogen interactions. Crucially, the physiological mechanisms enabling “high-yield and high-efficiency” populations to maintain source–sink coordination and resource balance under constrained water and nitrogen environments remain inadequately understood.

The interaction between root–canopy architecture and water–nitrogen utilization represents a critical link in yield formation for rainfed winter wheat [9]. Root systems enhance access to soil moisture and nutrients from soil layers [10,11], while higher leaf area index (LAI) improve light interception efficiency and sustain post-anthesis photosynthesis [12,13], thereby providing a stable source of assimilates for grain-filling. Different cultivars (types) exhibit significant differences in root distribution, canopy structure, patterns of dry matter partitioning, and nitrogen (N) remobilization capacity [14,15,16]. These differences subsequently drive variations in water use efficiency, nitrogen use efficiency, and yield stability. Consequently, the differences among cultivars in the proportions of pre- and post-anthesis dry matter accumulation (and their respective contributions to grain-filling), as well as in nitrogen remobilization efficiency, essentially reflect divergent source–sink regulation strategies and resource utilization patterns [17,18]. A detailed analysis of these key traits across different rainfed wheat types is crucial for elucidating the physiological regulatory characteristics of high-yield and high-efficiency populations, and it also provides a vital foundation for cultivar selection and the optimization of corresponding agronomic management practices.

This study employed multiple winter wheat cultivars adapted to rainfed systems, which were classified into four distinct groups based on grain yield and NUE: high-yield and high-efficiency (HH), high-yield and low-efficiency (HL), low-yield and high-efficiency (LH), and low-yield and low-efficiency (LL). Through a two-year field experiment, key traits across these groups were systematically analyzed, including yield components, grain-filling dynamics, patterns of dry matter accumulation and partitioning, water use dynamics, nitrogen uptake and remobilization, and root–canopy architecture. We hypothesize that high-yield and high-efficiency (HH) cultivars achieve superior performance through coordinated root–canopy structural traits that enhance water and nitrogen acquisition, maintain post-anthesis photosynthesis, and strengthen assimilate and nitrogen remobilization, thereby supporting greater sink capacity and kernel-filling. This study adopts an integrated multi-trait analytical framework, combining root architecture, canopy structure, water- and nitrogen-use characteristics, grain-filling processes, and biomass allocation to elucidate their collective contributions to yield and NUE differences among cultivar types. The primary objectives were (1) to compare the differential characteristics in water and nitrogen utilization and assimilate translocation among the diverse rainfed wheat types; (2) to elucidate the core physiological mechanisms underlying the formation of high-yield and high-efficiency populations; and (3) to provide theoretical foundations and practical guidance for achieving high-yield and high-efficiency production and cultivar improvement in rainfed wheat.

## 2. Results

### 2.1. Divergence in Yield Component Characteristics Among Winter Wheat Cultivar Types

**Figure 1:**
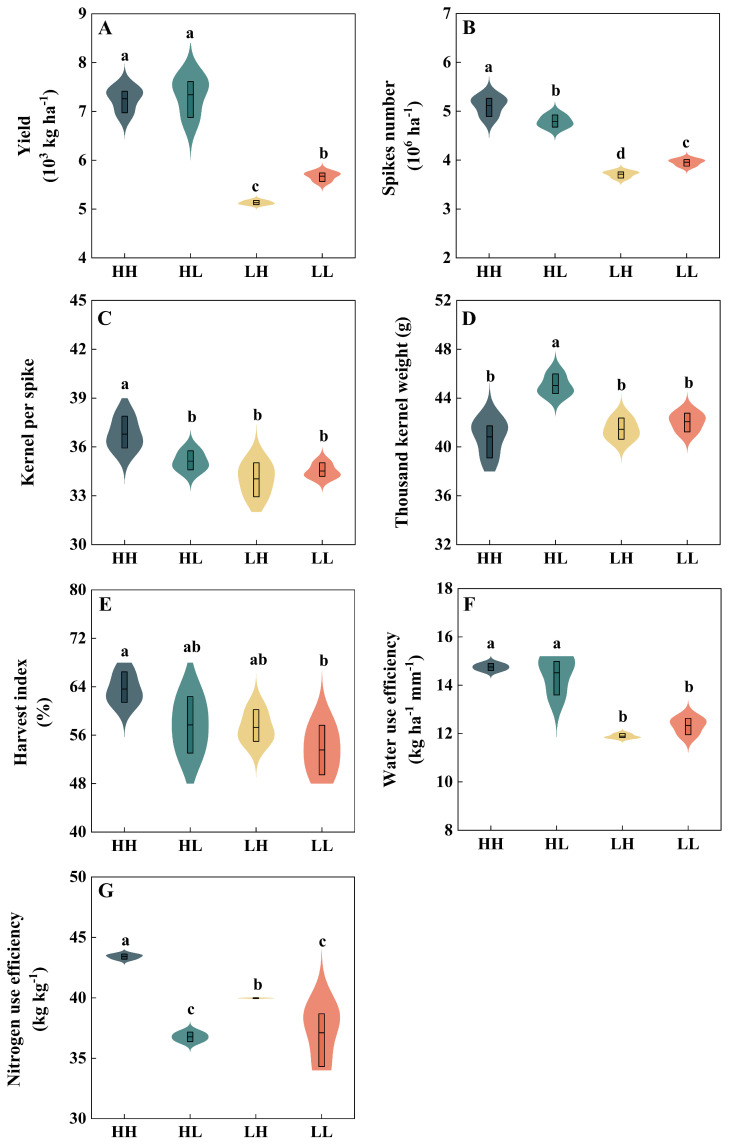
Differences in grain yield (**A**), spike number (**B**), kernel per spike (**C**), thousand-kernel weight (**D**), harvest index (**E**), WUE (**F**), and NUE (**G**) among winter wheat cultivar types (2022–2024). Different lowercase letters indicate statistically significant differences among cultivars (*p* < 0.05).

Compared with low-yield types (LH, LL), both HH and HL demonstrated significant advantages in grain yield and key agronomic traits. Specifically, relative to LH, HH and HL types’ yield increased significantly, by 41.5% and 43.1%, respectively, spike number increased by 37.8% and 29.1%, respectively, and the water use efficiency increased by 24.1% and 22.1%, respectively. Furthermore, kernels per spike were significantly increased by 8.1% in HH, while thousand-kernel weight was elevated by 8.6% in HL. When compared to LL, HH and HL showed superior performance across multiple parameters: grain yield increases of 27.9% and 29.4%, spike number increases of 29.3% and 21.1%, kernels per spike increases of 6.6% and 1.8%, harvest index improvements of 18.8% and 7.8%, and water use efficiency gains of 19.6% and 17.7%. Notably, thousand-kernel weight was also significantly enhanced by 7.0% in HL relative to LL. Moreover, the nitrogen use efficiency of the HH and LH types was significantly increased, by 18.1% and 8.7%, respectively, compared with HL, and by 17.0% and 7.7%, respectively, compared with LL (Figure 1).

### 2.2. Grain-Filling Kinetics

**Figure 2:**
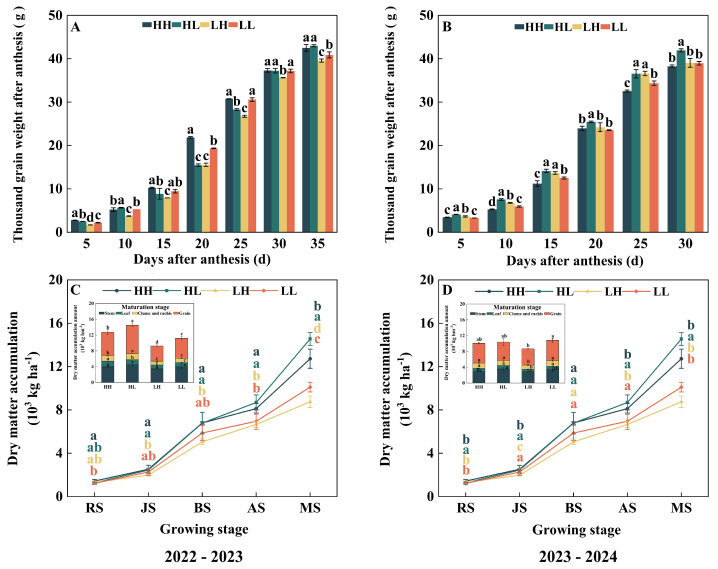
The differences in the dynamics of thousand-kernel weight after anthesis (**A**,**B**) and the dry matter accumulation of different types of wheat at different growth stage in dryland cultivation (**C**,**D**). Lowercase letters indicate significant differences among wheat types (*p* < 0.05). Within a column, different colored lowercase letters denote significant differences for the corresponding wheat type.

Thousand-kernel weight dynamics in diverse winter wheat types exhibited a typical “S” shaped growth trend with the increase in days after flowering (Figure 2A,B), with excellent fitness confirmed for the Logistic model (Table 1). In the 2022–2023 season, both HH and LL types achieved their maximum grain-filling rate earlier. Regarding grain-filling rates, compared to LH, the average grain-filling rate (Rmean) of HH increased significantly by 3.1%, and the fast-filling phase duration (t1), the slow-filling phase duration (t2) and time to maximum grain-filling rate (Tmax) were significantly increased by 6.2% to 12.5%. Conversely, t2, Tmax, Rmean, and the total grain-filling duration (T) of HL were significantly prolonged, by 4.1% to 9.5%, compared with LL, but the maximum grain-filling rate (Rmax) of HL was significantly decreased by 23.5%. Compared with LL, the T of HH was significantly prolonged by 6.0%, while Rmean decreased by 2.9%. Additionally, t1, t2, Tmax, and the T of HL increased by 13.9% to 18.1% compared with LL, but Rmax and Rmean were decreased by 25.9% and 1.9%, respectively.

During the 2023–2024 season, disparities between types narrowed notably for parameters including t2, Rmax, Tmax, and T. Within this year, t1 of HH and T of HL were significantly increased, by 5.7% and 8.2%, respectively, compared to LH, while the differences in other indicators were not significant. Compared with LL, the t1 of HL was significantly shortened by 5.0%, but its T value was significantly increased by 7.1%. Overall, variations in grain-filling progression among types were predominantly attributed to shifts in critical parameters such as t2, Tmax, and T (Table 1). Comparative analysis across two seasons revealed minimal divergence between curves during early grain-filling (5–15 days post-anthesis); however, in the middle and late grain-filling stages, a certain degree of differentiation became apparent, although its precise initiation point and magnitude varied interannually (Figure 2A,B).

### 2.3. Dry Matter Accumulation Characteristics and Organ Allocation Patterns

In both growing seasons, the dry matter accumulation of wheat above ground generally showed an increasing trend with the progression of the growth process. The periods from jointing to booting and from flowering to maturity were both stages of rapid dry matter accumulation, reaching a peak at maturity (Figure 2C,D). The results of the 2022–2023 growing season indicated that compared with LH, the above-ground dry matter weight of HH and HL significantly increased by 21.9–45.4% and 23.6–66.3%, respectively, during the jointing to maturity stage. Meanwhile, at the flowering and maturity stages, the above-ground dry matter of HH and HL was significantly higher than that of LL, with an increase of 16.7–44.0% (Figure 2C). In the 2023–2024 growing season, the dry matter accumulation of HH at the jointing stage significantly increased by 15.2% compared with LH. At the same time, the dry matter accumulation of HL-type wheat at the green returning, jointing, flowering, and maturity stages increased by 9.2–42.8% compared with LH. In addition, compared with LL, the dry matter accumulation of HL also showed a significant increase at the overwintering, green returning, and maturity stages, with an increase ranging from 13.0% to 38.8% (Figure 2D).

Further analysis of the distribution pattern of dry matter in different organs revealed that the allocation rules of various types of wheat remained consistent over the two years, with an overall performance as follows: grain > stem > glume + rachis > leaf. Comprehensive data from the two years showed that compared with low-yield types (LH and LL), high-yield types (HH and HL) allocated more dry matter to the grain at maturity (Figure 2C,D), reflecting better source–sink coordination ability. In 2022–2023, the dry matter accumulation of the grain, leaf, glume + rachis of high-yield types (HH and HL) was significantly higher than that of low-yield types (LH and LL), while there was no significant difference in the dry matter accumulation of stems among various types (Figure 2C). In 2023–2024, the dry matter accumulation of leaf and glume + rachis of high-yield types (HH and HL) was significantly higher than that of LH. At the same time, the dry matter accumulation of stem of HL was significantly increased by 25.8% compared with LH (Figure 2D). Overall, the advantages of high-yield groups (HH and HL) are mainly reflected in two aspects: higher total dry matter accumulation in the late growth stage and larger proportion allocated to the grain at maturity.

### 2.4. Soil Water Consumption Dynamic Characteristics

**Figure 3:**
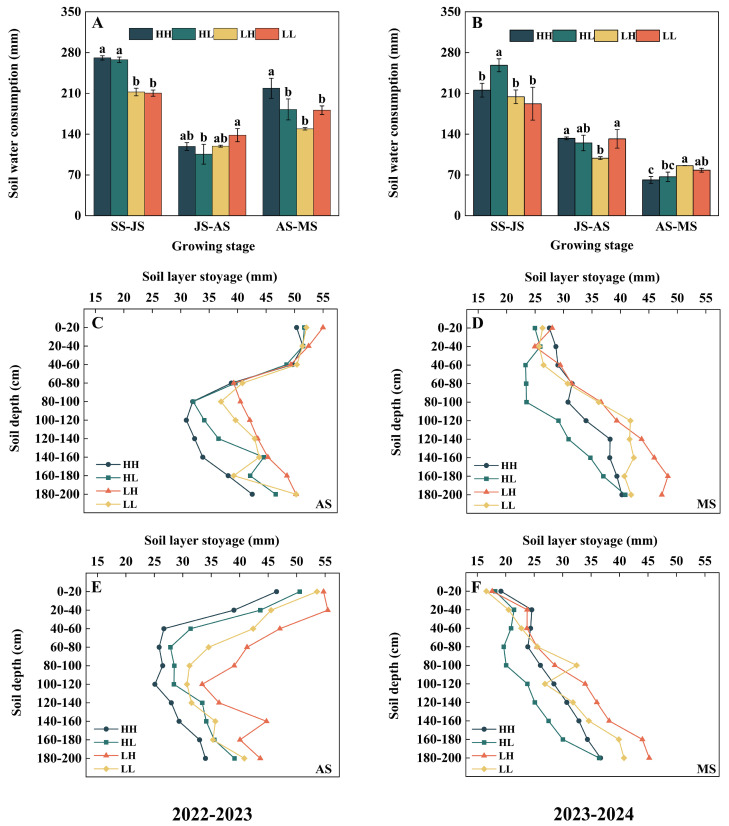
Differences in soil water consumption and soil layer water storage capacity during the two growing seasons. (**A**) soil water consumption in 2022–2023; (**B**) soil water consumption in 2023–2024; (**C**) Soil layer water storage capacity at anthesis stage in 2022–2023; (**D**) Soil layer water storage capacity at anthesis stage in 2023–2024; (**E**) Soil layer water storage capacity at maturity stage in 2022–2023; (**F**) Soil layer water storage capacity at maturity stage in 2023–2024. Different lowercase letters indicate statistically significant differences among cultivars (*p* < 0.05).

There were significant interannual differences in soil water consumption at various growth stages. In the 2022–2023 growing season, the dynamic pattern of water consumption throughout the whole growth period exhibited a three-stage characteristic of “high–low–high”. During the sowing to jointing stage, the water consumption of high-yield groups (HH and HL) was significantly higher than that of low-yield groups (LH and LL) by 27.2–28.7%, reflecting their high dependence on water for rapid growth in the early stage. Entering the flowering to maturity stage, the water consumption of the HH type further increased, being significantly higher than that of HL, LH, and LL by 19.9–46.7%, which indicated its strong water demand during the grain-filling period. Notably, during the jointing to flowering stage, the water consumption of HL was significantly reduced by 23.7% compared with LL (Figure 3A). In the 2023–2024 growing season, the stage-specific water consumption generally decreased gradually as the growth process advanced. Compared with low-yield groups (LH and LL), high-yield groups (HH and HL) had higher water consumption during the sowing to jointing and jointing to flowering stages, but lower water consumption during the flowering to maturity stage (Figure 3B). In conclusion, the advantages of high-yield groups (HH and HL) are mainly reflected in two aspects: one is the higher total dry matter accumulation in the late growth stage, and the other is the larger proportion allocated to the grain at maturity.

In both growing seasons, the soil water storage in the 0–200 cm soil layer exhibited obvious spatiotemporal heterogeneity with depth (Figure 3C–F). Generally speaking, the soil water storage of high-yield types (HH and HL) at the flowering and maturity stages was generally lower than that of low-yield types (LH and LL). In addition, during the flowering stage in both growing seasons, the water storage in the 0–200 cm soil layer of high-yield types (HH and HL) was lower than that of low-yield types (LH and LL).

### 2.5. Nitrogen Dynamic Characteristics

**Figure 4:**
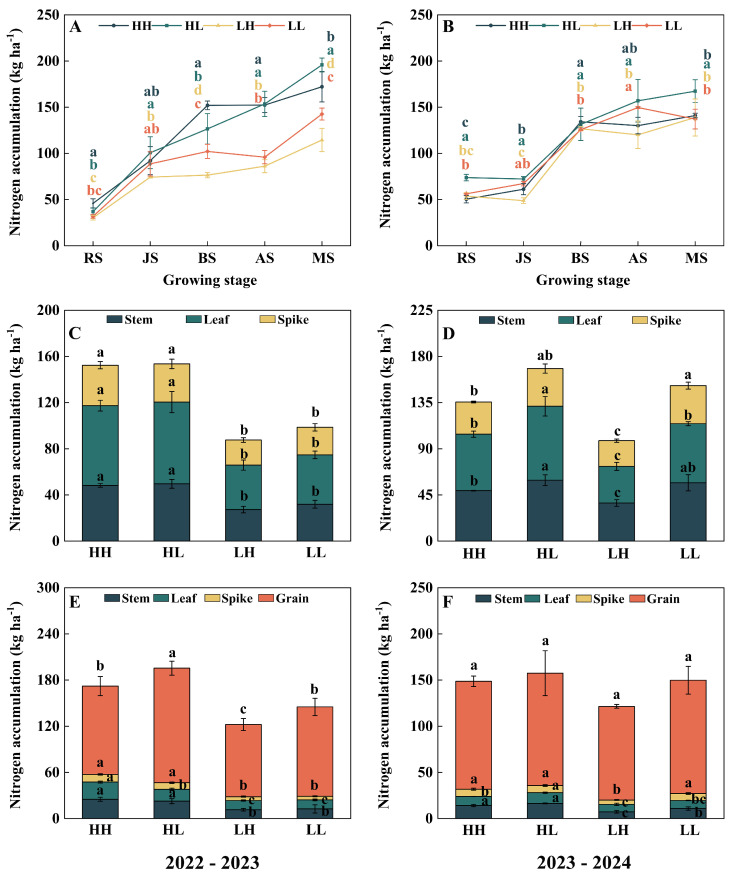
Differences in nitrogen accumulations of different types. (**A**) nitrogen accumulation of different growth stages in 2022–2023; (**B**) nitrogen accumulation of different growth stages in 2023–2024; (**C**) nitrogen accumulation at anthesis stage in 2022–2023; (**D**) nitrogen accumulation at anthesis stage in 2023–2024; (**E**) nitrogen accumulation at maturity stage in 2022–2023; (**F**) nitrogen accumulation at maturity stage in 2023–2024. Lowercase letters indicate significant differences among wheat types (*p* < 0.05). Within a column, different colored lowercase letters denote significant differences for the corresponding wheat type.

Overall, in both growing seasons, the aboveground nitrogen accumulation of all types of wheat plants continued to increase with the progression of the growth process (Figure 4A,B). In 2022–2023, the nitrogen accumulation of high-yield types (HH and HL) was significantly higher than that of low-yield ones (LH and LL) at most growth stages, with an increase range of 13.6% to 98.8%. However, no significant difference was observed between HL and LL at the green returning stage or between HH and LL at the jointing stage (Figure 4A). Entering the 2023–2024 growing season, the advantage of high-yield types in whole-plant nitrogen accumulation over low-yield types was more prominent. Although HH slightly decreased compared with LH at the returning green stage (a decrease of 6.2%), and decreased by 9.1% and 13.1%, respectively, compared with LL at the jointing and flowering stages, the overall trend still showed that high-yield varieties had stronger nitrogen accumulation ability (Figure 4B). Comprehensive data from two years shows that high-yield groups have a stable advantage in nitrogen uptake, while low-yield groups are generally at a lower level.

Further analysis of nitrogen distribution at the organ level revealed that during the two growing seasons, nitrogen in wheat plants was mainly concentrated in leaves and stems at the flowering stage, while grains became the primary storage site by maturity. Notably, at both the flowering and maturity stages across the two growing seasons, the nitrogen accumulation in leaves, stems, and spikes of high-yield cultivars (HH and HL) was significantly higher than that of low-yield type LH. Especially in 2022–2023, the advantage of high-yield types (HH and HL) in nitrogen accumulation in these organs was more prominent compared with LH. Meanwhile, the grain nitrogen accumulation of HH and LH at maturity was significantly increased by 22.3% and 58.6%, respectively, compared with LH, and that of HL was significantly increased by 28.2%. In the 2023–2024 growing season, high-yield types (HH and HL) still maintained an advantage in nitrogen accumulation over LH in most organs. Specifically, the nitrogen accumulation in leaves, stems, and spikes of high-yield wheat types (HH and HL) was significantly higher than that of LH at both the flowering and maturity stages. Compared with LL, HL showed a significant increase of 25.1–51.0% in leaf nitrogen accumulation at the flowering and maturity stages, as well as in stem nitrogen accumulation at maturity. At the same time, although HH had a significant increase of 29.8% in stem nitrogen accumulation at maturity, its spike nitrogen accumulation at the flowering stage decreased by 15.4% compared with LL. This reveals differences in nitrogen spatial distribution strategies among different high-yield types (Figure 4C–F).

### 2.6. Root–Canopy Structure

**Figure 5:**
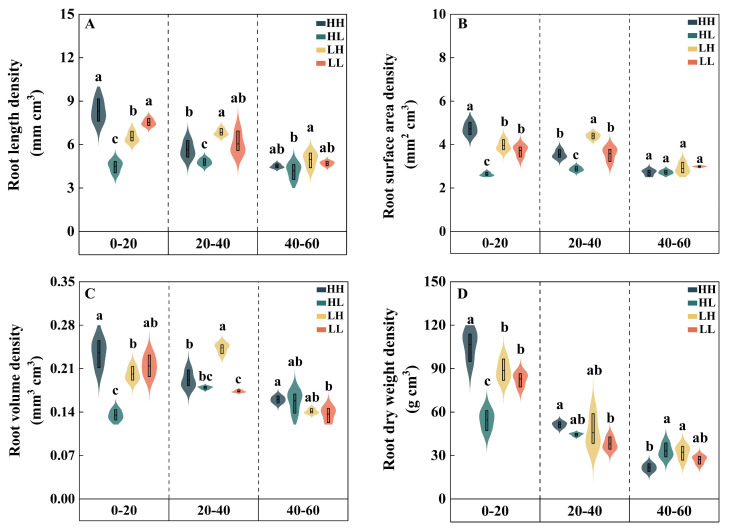
Differences in root morphological characteristics of different types of wheat in the 0–60 cm soil layer (2023–2024). (**A**) root length density; (**B**) root surface area density; (**C**) root volume density; (**D**) root dry weight density. Different lowercase letters indicate statistically significant differences among cultivars (*p* < 0.05).

At the flowering stage in the 0–20 cm soil layer, the root length density (RLD), root surface area density (RSD), root volume density (RVD), and root dry weight density (RDWD) of HH were significantly increased by 16.9% to 28.1% compared with LH. The RSD and RDWD of HH were significantly increased by 26.6% and 28.6%, respectively, compared with LL. Meanwhile, the RLD, RSD, and RVD of HL were significantly reduced by 30.7% to 33.5% compared with LH. The RSD, RVD, and RDWD of HL were significantly decreased by 29.5% to 37.0% compared with LL. In the 20–40 cm soil layer, the RLDs, RSDs, and RVDs of high-yield types (HH and HL) were significantly reduced by 17.5% to 34.6% compared with LH. The RLDs of HH and HL were significantly reduced by 6.7% and 20.8%, respectively, compared with LL. However, the RVD and RDWD of HH were significantly increased by 10.8% and 12.3%, respectively, compared with LL. At the same time, the RSD of HL was significantly decreased by 19.9% compared with LL. In the 40–60 cm soil layer, the RVD of HH was significantly increased by 17.1% compared with LL, while the RDWD of HH was significantly decreased by 32.9% compared with LH. The RLD of HL was significantly decreased by 15.8% compared with LH (Figure 5).

**Figure 6:**
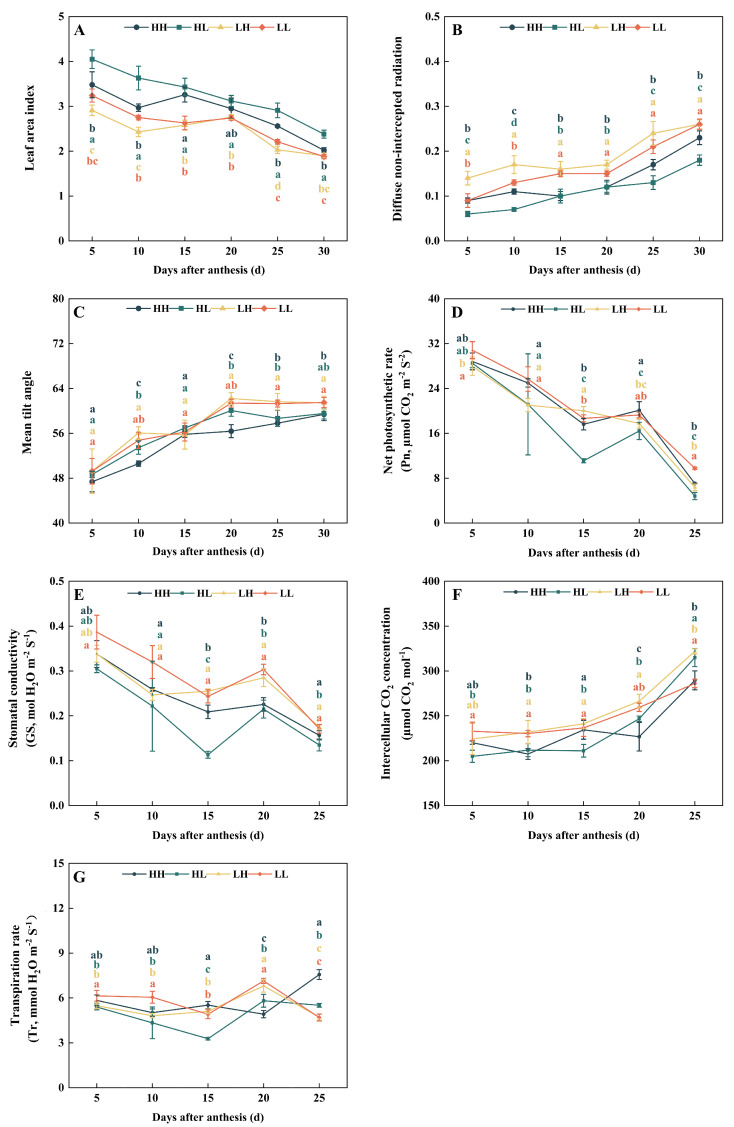
Differences in photosynthetic characteristics among different types (2023–2024). (**A**) leaf area index; (**B**) the diffuse non-intercepted radiation; (**C**) mean tilt angle; (**D**) net photosynthetic rate; (**E**) stomatal conductance; (**F**) intercellular CO_2_ concentration; (**G**) transpiration rate. Lowercase letters indicate significant differences among wheat types (*p* < 0.05). Within a column, different colored lowercase letters denote significant differences for the corresponding wheat type.

Further analysis of the wheat canopy revealed that, overall, the leaf area index gradually decreased as the growth process advanced, while the diffuse non-intercepted radiation (DIFN) and mean tilt angle (MTA) gradually increased (Figure 6A–C). From 5 to 30 days after anthesis, the leaf area index of high-yield types (HH and HL) was higher than that of low-yield types (LH and LL) by 6.3% to 49.4%. Meanwhile, the hierarchical levels between different types remained stable at most time points. However, DIFN and MTA showed opposite trends. The DIFN and MTA of high-yield types (HH and HL) were lower than those of low-yield types (LH and LL) by 11.5% to 58.8% (except at 5 days after anthesis) and 1.2% to 9.8% (except at 15 days after anthesis), respectively, at multiple time points from 5 to 30 days after anthesis. This indicates that the canopy of high-yield types is more compact (with a smaller openness and smaller leaf inclination angle) compared with that of low-yield types, which may be more conducive to the distribution of light energy within the population and the coordinated assimilation of upper- and middle-layer leaves.

Analysis of photosynthetic characteristics revealed that with the progression after anthesis, the net photosynthetic rate, stomatal conductance, and transpiration rate of flag leaves of different types of wheat generally showed a trend of first decreasing, then increasing, and then decreasing again, while the intercellular carbon dioxide concentration gradually increased. The net photosynthetic rate, stomatal conductance, transpiration rate, and intercellular carbon dioxide concentration in flag leaves of high-yield types (HH and HL) were lower than those of low-yield types (LH and LL). The transpiration rate of HH was significantly higher than that of low-yield types (LH and LL) at 15 and 25 days after anthesis, with an increase ranging from 8.2% to 61.2%. At the same time, at 25 days after anthesis, the transpiration rate of low-yield types (LH and LL) showed a downward trend, while that of HH showed an upward trend, which may indicate the existence of other photosynthetic characteristics (Figure 6D–G).

### 2.7. Analysis of the Association Between Factors Influencing Wheat Yield Formation

**Figure 7:**
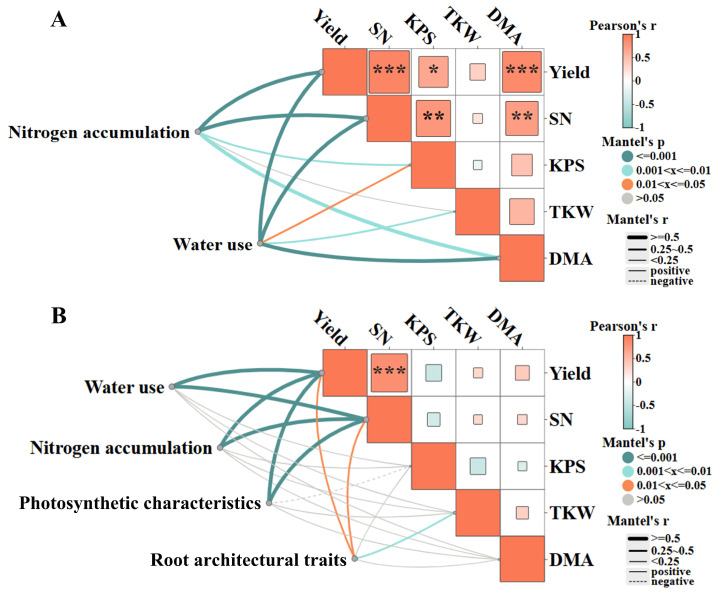
The associations between wheat growth performance indicators and nitrogen accumulation, water use, photosynthetic characteristics, and root architectural traits. Wheat growth performance indicators (SN, KPS, TKW, DMA represent spike number, kernels per spike, thousand-kernel weight, and dry matter accumulation, respectively) and their associations with other characteristics. (**A**) The associations of this set of indicators with nitrogen accumulation and water use from 2022 to 2024. (**B**) This part presents more in-depth associations after incorporating photosynthetic characteristics and root architectural traits in 2023–2024. * *p* < 0.05; ** *p* < 0.01; *** *p* < 0.001, n = 12.

Based on the association analysis of wheat growth performance with nitrogen accumulation, water use, photosynthetic characteristics, and root architectural traits (Figure 7). From 2022 to 2024, there was a significant positive correlation between wheat yield and spike number, kernels per spike, and dry matter accumulation (*p* < 0.05, *p* < 0.001). Meanwhile, spike number had an extremely significant positive correlation with kernels per spike and dry matter accumulation (*p* < 0.01). Nitrogen accumulation and water use both had a significant positive correlation with yield, spike number, kernels per spike, and dry matter accumulation (*p* < 0.05, *p* < 0.01, *p* < 0.001). At the same time, water use and thousand-kernel weight had an extremely significant positive correlation (*p* < 0.01) (Figure 7A). In 2023–2024, there was an extremely significant positive correlation between yield and spike number (*p* < 0.001). Meanwhile, nitrogen accumulation, water use, photosynthetic characteristics, and root architectural traits all had a significant positive correlation with yield and spike number (*p* < 0.05, *p* < 0.001), and root architectural traits also had an extremely significant positive correlation with thousand-kernel weight (*p* < 0.01) (Figure 7B).

## 3. Discussion

This study reveals that high-yield groups, particularly the HH type, exhibit advantages in multiple aspects, such as pre-flower reserves, post-flower assimilation, and material transportation. This indicates that the formation of yield depends on a complete “source–sink–flow” coupling system [19]. Crucially, under water constraints, when post-flowering assimilation is inhibited, the mobilization and redistribution of pre-anthesis reserved photoassimilates become paramount for sustaining grain-filling [20]. This study shows that in relatively dry years, the contribution of pre-flower transport to grains is higher; in years with more rainfall, the weight of post-flower nitrogen absorption and assimilation increases. For instance, adequate anthesis-period precipitation during 2022–2023 facilitated greater post-anthesis dry matter accumulation compared to the markedly drier 2023–2024 season. This phenomenon, “annual precipitation-driven dry matter partitioning adjustment” aligns with previous findings: post-anthesis water stress frequently triggers premature senescence, diminishes photosynthesis, and abbreviates the grain-filling phase, consequently increasing the mobilization of pre-anthesis reserves; conversely, favorable hydrothermal regimes enhance the contribution of post-anthesis assimilation and nitrogen acquisition to grain plumpness [20,21].

HH types further exhibited superior efficiency in nitrogen uptake and allocation. Data from both growing seasons revealed that the HH and HL types achieved significantly higher above-ground nitrogen accumulation at most developmental stages compared to low-yield counterparts. Critically, these high-yield types demonstrated an enhanced capacity for nitrogen remobilization, from vegetative tissues to the grain during grain-filling, resulting in a greater proportion of total plant nitrogen being partitioned to the grain at maturity. These findings indicate that high-yield cultivars can meet the nitrogen demands during grain-filling by means of stem nitrogen remobilization, thereby achieving a dynamic source–sink balance [22]. Additionally, it is worth noting that although agronomic NUE was used as the basis for cultivar classification, several physiological processes, including nitrogen uptake, redistribution, and canopy function, collectively contributed to the observed efficiency differences [23].

This study demonstrates that HH wheat optimizes physiological performance through the synergistic coupling of root system architecture and canopy photosynthetic characteristics, establishing a critical foundation for maintaining stable carbon and nitrogen supply during grain-filling. Regarding root traits, HH types exhibited significantly higher root length density, root surface area density, root volume density, and root dry-weight density in the shallow soil layer (0–20 cm) compared to other groups. This indicates a strategic prioritization of developing dense, superficial root systems under rain-fed conditions, facilitating the efficient capture of water from ephemeral rainfall. This is a strategy widely recognized as vital for early population establishment and water acquisition in dryland wheat systems [10,24]. Concurrently, HH maintained a certain number of roots in deeper soil layers, providing the compensatory water absorption capacity essential for sustaining late-season photosynthesis and nitrogen remobilization. In contrast, the HL type displayed deficiencies in both shallow and medium-depth roots, likely constraining its access to water and nitrogen during later developmental stages, thereby resulting in a relatively low NUE level.

In concert with its root system architecture, the canopy characteristics of HH established a relatively efficient population photosynthetic system. During the post-anthesis phase, HH exhibited a higher LAI coupled with reduced DIFN and MTA, resulting in a more compact canopy structure with pronounced vertical stratification. While elevated LAI generally risks uneven light distribution within dense canopies, potentially diminishing unit-leaf photosynthetic efficiency; this is particularly problematic under drought conditions, where upper-canopy light saturation and lower-canopy shading frequently occur [25,26]. Nevertheless, our study demonstrated that HH maintained high net photosynthetic rates in leaves during mid-to-late grain-filling. Remarkably, this performance persisted despite the denser canopy and increased LAI, showing no significant signs of photosynthetic limitation. Consequently, HH sustained a comparatively high population photosynthetic assimilation capacity. Supporting evidence from recent research confirms that such compact wheat canopies enhance the vertical profile of light interception, thereby improving light use efficiency in middle-layer leaves [26,27].

High-yield types, particularly HH wheat, demonstrated superior coordination between population size and individual plant yield. Fundamentally, the higher yield of the HH type was driven by greater sink capacity, reflected in a higher grain number per unit area derived from both increased spike number and improved kernel set. Both HH and HL types exhibited significantly more spikes than low-yield types. Concurrently, HH excelled in kernels per spike while HL showed advantages in thousand-kernel weight. This indicates that high-yield types do not rely solely on larger population sizes; rather, they maintain effective grain-filling and seed plumpness even with a “multi-spike” architecture, achieving simultaneous improvement in both population-level and single-plant yield [28]. This characteristic of population–individual synergy is linked to their root–canopy structural advantages. Their well-developed shallow root systems, combined with compact, efficient canopies, enhanced early population establishment. Furthermore, these structures provided stable photosynthetic capacity and water–nitrogen supply during later developmental stages, supporting the assimilate demands of increased spike numbers while ensuring adequate grain-filling for kernel weight. Consequently, high-yield types achieved a balance between population quantity and individual productivity [29], reinforcing their capacity for stable, high-yield performance.

Collectively, high-yield cultivars possess structural advantages in shallow root systems and compact canopies, alongside superior coordination between population size and individual plant yield. A higher number of kernels per spike provides the population size foundation for yield. On this basis, it is still possible to maintain a large number of kernels per spike or a high thousand-kernel weight, indicating that the photosynthetic substances and water–nutrient supply can simultaneously meet the needs of more spike positions and fullness of the grains. The optimized root–canopy architecture enhances early population establishment [30] and sustains stable assimilate supply during later developmental stages. This enables high-yield types to achieve a critical balance between population quantity and individual kernel weight, constituting an important mechanism underpinning their stable high-yield performance.

Significant differences existed in water consumption patterns among winter wheat types, profoundly impacting carbon supply during grain-filling and water use efficiency. This study revealed that in the wet year (2022–2023), high-yield groups, particularly the HH type, exhibited substantially higher water consumption than low-yield types during the sowing–jointing phase. This enabled them to rapidly establish a substantial biomass base early in population development. Such an “early high water consumption–rapid population growth” strategy, beneficial for expanding source–sink capacity, has been documented in some studies to enhance post-anthesis photosynthetic assimilation in dryland wheat [31]. Conversely, in the drought year (2023–2024), HH demonstrated lower water usage during anthesis–maturity compared to low-yield types, which was concomitant with higher WUE. This indicates that HH possesses the capacity to flexibly modulate its water consumption rhythm according to water availability: enhancing source–sink scale via increased transpiration and assimilation when water is ample, yet minimizing carbon loss by reducing late-stage transpiration under water-limited conditions, thereby sustaining grain-filling supply [32].

The overall water storage in the 0–200 cm soil layer of HH at flowering and maturity stages was lower than that of low-yield types, indicating that it makes more thorough use of soil moisture. Previous studies have suggested that cultivars capable of accessing greater water reserves from mid-depth soil layers are better positioned to sustain post-anthesis photosynthetic assimilation during the later stages of grain-filling [33,34]. In this study, HH’s maintenance of a relatively high root volume density at certain depths may also facilitate the mobilization of deeper soil water reserves during mid-to-late development, thereby supporting photosynthetic activity and nitrogen remobilization throughout grain-filling.

Collectively, HH demonstrates environment-dependent water use strategies: adopting a “high water expenditure for vigorous growth” approach in wet years while shifting towards “conserved water use for efficiency maintenance” in drought years. This adaptive plasticity enables sustained source-sink capacity under both conditions. Crucially, such strategic plasticity underpins relatively stable yield performance across seasons with highly variable rainfall patterns in dryland farming systems.

## 4. Materials and Methods

### 4.1. Experimental Material

Ten winter wheat cultivars adapted to rainfed systems were selected for this study: Chang 6359, Linhan 8, Zhongmai 36, Yunhan 20410, Luohan 6, Jinmai 92, Yunhan 618, Jinmai 47, Yunhan 115, and Yunhan 805 (Table S1). The ten cultivars were selected because they are widely grown or regionally representative of dryland wheat production on the Loess Plateau and also exhibit contrasting yield and nitrogen-use efficiency levels based on long-term regional trials. This diversity allowed us to capture a wide physiological range and to meaningfully compare different performance types (HH, HL, LH, LL).

### 4.2. Experiment Design

The field experiment was conducted during two consecutive growing seasons (2022–2023 and 2023–2024) at the Wheat Experimental Base of Shanxi Agricultural University, located in the rainfed agricultural region of the eastern Loess Plateau, Shanxi Province, China (34°35′ N, 110°15′ E). The soil type of the experimental field is calcareous cinnamon soil, classified according to the USDA soil taxonomy system. Pre-sowing soil nutrient indicators were all assayed following Bao’s method [35]: active acidity (pH) was measured by the soil–water extraction-pH meter (1:2.5 ratio, 1:5 for organic soils); organic content was measured by potassium dichromate volumetry; alkaline hydrolyzable nitrogen content was measured by alkaline hydrolysis diffusion; available phosphorus content was measured by NaHCO_3_ extraction–molybdenum blue colorimetry; available potassium content was measured by NH_4_OAc extraction–flame photometry; and total nitrogen content was measured by the Kjeldahl method. Seasonal precipitation totals are provided in Figure S1. Basic soil fertility parameters (0–20 cm and 20–40 cm depths) prior to sowing are summarized in Table 2. Each wheat variety was grown in three replicate plots (6 m × 6 m) arranged in a completely randomized block design. No irrigation was applied during the fallow period. Compound fertilizer (N:P_2_O_5_:K_2_O = 21:17:6), with nitrogen sourced from ammonium sulfate, was applied as a single basal dose before sowing, equivalent to 180 kg N ha^−1^. No additional topdressing fertilizer or irrigation was applied throughout the growing season. A skip-row planting pattern with wide rows (20–25 cm) alternated with narrow rows (10–12 cm) was used. Wheat was sown on 28 September 2022 and 10 October 2023, at seeding rates of 180 kg ha^−1^ and 240 kg ha^−1^, respectively, and harvested on 9 June 2023 and 3 June 2024, respectively.

### 4.3. Dry Matter Accumulation and Translocation

In each growing season, above-ground plant samples were randomly collected from 20 plants per cultivar at the following key growth stages: returning green stage (RS; 28 September 2022 and 8 October 2023), jointing stage (JS; 21 March 2023 and 29 March 2024), booting stage (BS; 14 April 2023 and 17 April 2024), anthesis stage (AS; 30 April 2023 and 24 April 2024), and maturity stage (MS; 9 June 2023 and 30 May 2024). At the returning green and jointing stages, whole plants were sampled. At the booting stage, plants were separated into leaf and stem. At the anthesis stage, plants were divided into leaf, stem, and spike. At maturity, plants were partitioned into leaf, stem, glume + rachis, and grain. All separated plant components were placed in kraft paper bags, heated at 105 °C for 30 min, and then further dried at 80 °C to a constant weight before weighing. Three biological replicates were performed for each cultivar. The following parameters were calculated: pre-anthesis dry matter translocation, pre-anthesis dry matter translocation efficiency, contribution of pre-anthesis dry matter translocation to grain yield, post-anthesis dry matter accumulation, and contribution of post-anthesis dry matter production to grain yield [36].

### 4.4. Grain Yield and Its Components

At maturity, spike numbers were counted within three randomly selected 0.667 m^2^ fixed quadrats per plot, and grains were collected. Concurrently, twenty uniform spikes were randomly sampled from each plot to determine total kernels per spike, which was used to calculate kernels per spike. After drying, thousand-kernel weight was measured. Grain yield and moisture content were recorded from a randomly selected 0.667 m^2^ area within each plot. Actual yield was standardized to a moisture content of 13% according to the national grain storage standard.

### 4.5. Grain Filling

In both growing seasons, ten uniform plants per cultivar plot were sampled at 5-day intervals from 5 to 35 days after anthesis in the first season and from 5 to 30 days in the second season. The threshed grains were dried and weighed. The grain-filling process was fitted using a logistic growth model [37].

### 4.6. Water Determination

Soil samples were collected at sowing stage (SS), jointing stage, anthesis stage, and maturity stage using a soil auger for depth-specific sampling. The profile was sampled to a depth of 200 cm, divided into 20 cm increments. Samples from each layer were placed into pre-weighed and numbered aluminum containers. Fresh weight was measured using an electronic balance (precision: 0.01 g) and recorded. Subsequently, the containers with soil were oven-dried at 105 °C to constant weight, and dry weight was measured. Three biological replicates were performed. Water consumption, water storage, and water use efficiency were calculated based on these measurements [38,39].

The soil water storage capacity for each soil layer is calculated using the following formula:

SWS_i_ = W_i_ × D_i_ × H_i_ × 10/100 (1)

where SWS denotes the water storage capacity (mm), i denotes the soil layer, W denotes the soil moisture content (%), D denotes the soil density (g cm^−3^), and H denotes the thickness of the soil layer (cm).

ET = ΔS + P (2)

∆S = S1 − S2 (3)

where ET denotes armland soil water consumption (mm); ΔS denotes the change in soil water storage, with S1 and S2 denoting the soil water storage (mm) at the beginning and end of the growth stage, respectively; P denotes the effective precipitation (mm) during the cropping season.

WUE = Y/ET (4)

where WUE denotes water use efficiency (kg hm^−2^ mm^−1^); Y denotes wheat grain yield.

### 4.7. Nitrogen Accumulation, Transport and Nitrogen Use Efficiency

At the flowering and maturity stages, dried samples of each organ were ground in a grinder. The nitrogen content of the plants was then determined using the H_2_SO_4_-H_2_O_2_-indophenol blue colorimetric method [40], and the nitrogen accumulation and nitrogen use efficiency were calculated [38].

The nitrogen-related calculation formulas used in this study are as follows:

Nitrogen use efficiency (NUE, kg kg^−1^) = Grain yield (kg hm^−2^)/above-ground nitrogen accumulation (kg hm^−2^) (5)

Plant nitrogen accumulation (kg hm^−2^) = plant dry matter weight × nitrogen concentration (6)

Pre-anthesis nitrogen translocation amount (kg hm^−2^) = above-ground nitrogen accumulation at anthesis (kg hm^−2^) − nitrogen accumulation in non-grain organs at maturity (kg hm^−2^) (7)

Pre-anthesis nitrogen translocation rate (%) = (pre-anthesis nitrogen translocation amount (kg hm^−2^)/above-ground nitrogen accumulation at anthesis (kg hm^−2^)) × 100% (8)

Contribution rate of pre-anthesis nitrogen translocation to grain nitrogen accumulation (%) = (pre-anthesis nitrogen translocation amount (kg hm^−2^)/grain nitrogen accumulation at maturity (kg hm^−2^)) × 100% (9)

Post-anthesis nitrogen accumulation (kg hm^−2^) = grain nitrogen accumulation at maturity (kg hm^−2^) − pre-anthesis nitrogen translocation amount (kg hm^−2^) (10)

Contribution rate of post-anthesis nitrogen uptake to grain nitrogen accumulation (%) = (post-anthesis nitrogen uptake (kg hm^−2^)/grain nitrogen accumulation at maturity (kg hm^−2^)) × 100% (11)

### 4.8. Root Morphological Traits

During the 2023–2024 growing season, root samples were collected at anthesis using a root auger (10 cm diameter, 20 cm length). Soil cores were taken to a depth of 0.6 m, with three replicates per plot. Root samples were soaked in a 100 mesh nylon net for 1 h, rinsed with clean water to remove debris, and stored. Roots were scanned using a root scanner, and root length, surface area, and volume were analyzed. Subsequently, roots were oven-dried at 75 °C to constant weight, and dry weight was recorded. Root length density, root surface area density, root volume density, and root dry weight density were calculated [41].

### 4.9. Canopy and Photosynthetic Characteristics

During the 2023–2024 growing season, from 5 to 30 days after anthesis at 5-day intervals, five to ten uniform flag leaves per plot were tagged. Between 9:00 AM and 11:00 AM, canopy characteristics including leaf area index, diffuse non-intercepted radiation, and mean tilt angle were measured using an LAI-2200C plant canopy analyzer (LI-COR Biosciences, Lincoln, NE, USA). Concurrently, the net photosynthetic rate (Pn), stomatal conductance (GS), intercellular CO_2_ concentration (Ci), and transpiration rate (Tr) of the tagged flag leaves were determined using an LI-6400XT portable photosynthesis system (LI-COR Biosciences, Lincoln, NE, USA).

### 4.10. Data Analysis

#### 4.10.1. Variety Classification

Based on the clustering of two seasons’ yields and nitrogen use efficiency, the grain yield and nitrogen use efficiency data indicators were placed on the coordinate axes as the *x*-axis and *y*-axis coordinates, with their average values serving as the starting points of the coordinate axes. This divided the ten dryland wheat cultivars into four types. C 6359, LH 8, and ZM 36 were classified as the high-yield and high-efficiency type. LH 6 and YH 20,410 were categorized as the high-yield and low-efficiency type. YH 618 and JM 92 were designated as the low-yield and high-efficiency type. YH 805, YH 115, and JM 47 were grouped as the low-yield and low-efficiency type (Figure S2).

#### 4.10.2. Statistical Analysis

Data entry and organization were performed using Microsoft Excel 2019 (Microsoft Corporation, Redmond, WA, USA). SPSS version 28.0 software (IBM Corporation, Armonk, NY, USA) was used for multi-comparison analysis among different groups (LSD test, *p* < 0.05). Data visualization was conducted using Origin 2024 (OriginLab Corporation, Northampton, MA, USA). Grain-filling curves with R^2^ < 0.90 were subjected to re-evaluation. The ChiPlot online platform (https://www.chiplot.online/, accessed on 28 December 2025) was employed to perform Mantel tests. These analyses examined the relationships between: nitrogen accumulation, water dynamics, and wheat growth indicators for the 2022–2024 period; and nitrogen accumulation, water dynamics, photosynthetic characteristics (including canopy properties), root morphological traits, and wheat growth indicators for the 2023–2024 period. Correlation matrices were visualized using this platform.

## 5. Conclusions

This study systematically compared differences in root–canopy architecture, water and nitrogen utilization, dry matter accumulation, and remobilization among diverse types of winter wheat grown in dryland systems. The results demonstrated that the HH type (ZM36, LH8 and C6359) achieved more efficient water uptake, photosynthetic maintenance, and nitrogen remobilization capacity by developing shallow-rooted yet moderately deep-penetrating root systems coupled with compact canopies exhibiting high leaf area index. Concurrently, HH sustained the prolonged translocation of both dry matter and nitrogen to grains during late development, enhancing source–sink coordination throughout grain-filling. This enabled substantial spike numbers without compromising kernel plumpness. Collectively, through optimized root–canopy synergy, efficient water–nitrogen resource use, and balanced population–individual productivity, HH genotypes established stable high-yield performance with superior resource use efficiency under rain-fed conditions. These findings provide critical theoretical foundations for winter wheat cultivar selection and highly efficient cultivation techniques in arid agroecosystems.

## Figures and Tables

**Table 1:** Differences in grain=filling model parameters of different dryland wheat in two growing seasons.

Year	Varieties Type	t1 (d)	t2 (d)	Rmax (g 1000-Grain^−1^ d^−1^)	Tmax (d)	Rmean (g 1000-Grain^−1^ d^−1^)	T (d)	R^2^
2022–2023	HH	14.11 ± 0.15 b	24.30 ± 0.11 c	2.14 ± 0.04 ab	20.67 ± 0.12 c	1.00 ± 0.01 b	44.72 ± 0.01 b	0.998
	HL	16.24 ± 0.06 a	27.42 ± 0.21 a	1.69 ± 0.71 b	23.43 ± 0.11 a	1.01 ± 0.01 b	49.82 ± 0.75 a	0.996
	LH	16.13 ± 0.23 a	25.90 ± 0.54 b	2.21 ± 0.02 a	22.42 ± 0.44 b	0.97 ± 0.01 c	45.48 ± 0.84 b	0.998
	LL	14.26 ± 0.21 b	23.55 ± 0.11 c	2.28 ± 0.05 a	20.24 ± 0.15 c	1.03 ± 0.01 a	42.19 ± 0.09 c	0.998
2023–2024	HH	13.31 ± 0.16 a	22.43 ± 0.53 a	2.32 ± 0.22 a	19.18 ± 0.31 a	1.06 ± 0.08 a	40.71 ± 1.25 b	0.998
	HL	12.39 ± 0.27 b	22.97 ± 0.22 a	2.14 ± 0.17 a	19.20 ± 0.19 a	1.05 ± 0.06 a	44.19 ± 0.51 a	0.998
	LH	12.59 ± 0.36 b	21.98 ± 0.82 a	2.29 ± 0.04 a	18.64 ± 0.66 a	1.08 ± 0.02 a	40.83 ± 1.23 b	0.991
	LL	13.04 ± 0.14 a	22.43 ± 0.50 a	2.29 ± 0.10 a	19.08 ± 0.36 a	1.07 ± 0.04 a	41.25 ± 0.94 b	0.998

Note: t1, t2, Rmax, Tmax, Rmean, and T denote the fast-filling phase duration, slow-filling phase duration, maximum grain-filling rate, time to maximum grain-filling rate, average grain-filling rate, and total grain-filling duration, respectively. Data in the table are average value ± standard deviation. Different lowercase letters in the same column indicate significant differences among cultivars (*p* < 0.05).

**Table 2:** The soil basic fertility of the experimental field before sowing in 2022.

Soil Depth	pH	Organic Content (g kg^−1^)	Alkali Hydrolyzable Nitrogen Content (mg kg^−1^)	Available Phosphorus Content (mg kg^−1^)	Available Potassium Content (mg kg^−1^)	Total Nitrogen Content (g kg^−1^)
0–20	7.93	8.72	33.33	7.5	119.33	0.67
20–40	7.92	7.49	28.14	12.53	115	0.63

## Data Availability

The original contributions presented in this study are included in the article/Supplementary Materials. Further inquiries can be directed to the corresponding author.

## Supplementary Materials

The supporting information can be downloaded at: https://www.mdpi.com/article/10.3390/plants15010153/s1.

## Abbreviations

The following abbreviations are used in this manuscript:

HH	High-yield and high-efficiency
HL	High-yield and low-efficiency
LH	Low-yield and high-efficiency
LL	Low-yield and low-efficiency
WUE	Water use efficiency
NUE	Nitrogen use efficiency
Rmean	Average grain-filling rate
t1	The fast-filling phase duration
t2	The slow-filling phase duration
Tmax	Time to maximum grain-filling rate
T	The total grain-filling duration
Rmax	Maximum grain-filling rate
RLD	Root length density
RSD	Root surface area density
RVD	Root volume density
RDWD	Root dry weight density
LAI	Leaf area index
DIFN	Diffuse non-intercepted radiation
MTA	Mean tilt angle
SN	Spike number
KPS	Kernels per spike
TKW	Thousand-kernel weight
DMA	Dry matter accumulation
SS	Sowing stage
RS	Returning green stage
JS	Jointing stage
BS	Booting stage
AS	Anthesis stage
MS	Maturity stage
Pn	Net photosynthetic rate
GS	Stomatal conductance
Ci	Intercellular CO_2_ concentration
Tr	Transpiration rate
