# Supplementary material for: Root–Canopy Coordination Drives High Yield and Nitrogen Use Efficiency in Dryland Winter Wheat
Source: Plants (Basel). 2026 Jan 4;15(1):153. doi: 10.3390/plants15010153 (PMC12787808; doi:10.3390/plants15010153)
Supplement: Supplementary file 1 [file plants-15-00153-s001.zip › Supplementary Figure.pdf]

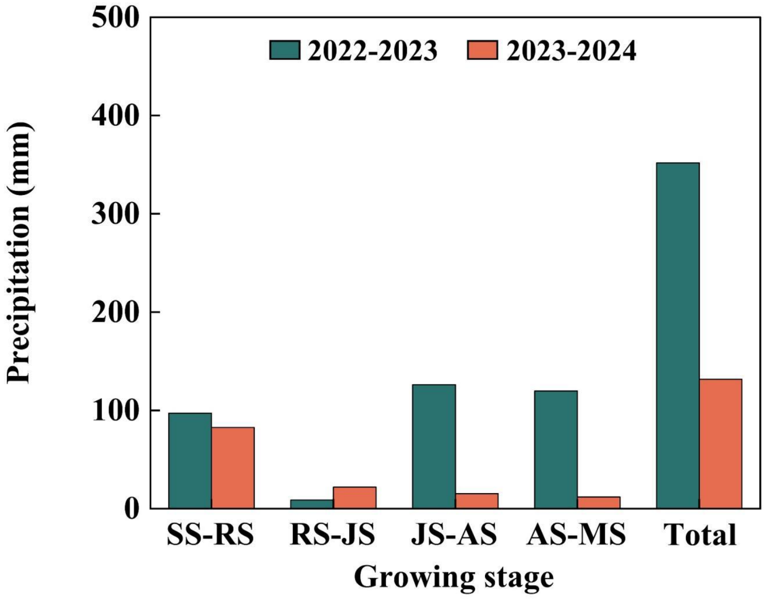

Figure S1. Test point rainfall (2022–2024).

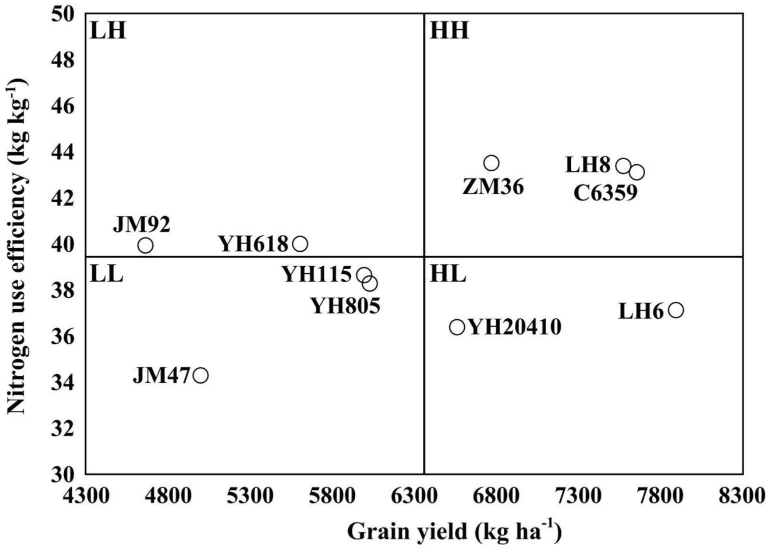

Figure S2. Distribution patterns of grain yield and nitrogen use efficiency among dryland wheat cultivars (2022–2024).
